# Shining light on the head: Photobiomodulation for brain disorders

**DOI:** 10.1016/j.bbacli.2016.09.002

**Published:** 2016-10-01

**Authors:** Michael R. Hamblin

**Affiliations:** Wellman Center for Photomedicine, Massachusetts General Hospital, Boston, MA 02114, USA; Department of Dermatology, Harvard Medical School, Boston, MA 02115, USA; Harvard-MIT Division of Health Sciences and Technology, Cambridge, MA 02139, USA

**Keywords:** Photobiomodulation, Low level laser (light) therapy, Ischemic stroke, Traumatic brain injury, Alzheimer's disease, Parkinson's disease, Major depression, Cognitive enhancement

## Abstract

Photobiomodulation (PBM) describes the use of red or near-infrared light to stimulate, heal, regenerate, and protect tissue that has either been injured, is degenerating, or else is at risk of dying. One of the organ systems of the human body that is most necessary to life, and whose optimum functioning is most worried about by humankind in general, is the brain. The brain suffers from many different disorders that can be classified into three broad groupings: traumatic events (stroke, traumatic brain injury, and global ischemia), degenerative diseases (dementia, Alzheimer's and Parkinson's), and psychiatric disorders (depression, anxiety, post traumatic stress disorder). There is some evidence that all these seemingly diverse conditions can be beneficially affected by applying light to the head. There is even the possibility that PBM could be used for cognitive enhancement in normal healthy people. In this transcranial PBM (tPBM) application, near-infrared (NIR) light is often applied to the forehead because of the better penetration (no hair, longer wavelength). Some workers have used lasers, but recently the introduction of inexpensive light emitting diode (LED) arrays has allowed the development of light emitting helmets or “brain caps”. This review will cover the mechanisms of action of photobiomodulation to the brain, and summarize some of the key pre-clinical studies and clinical trials that have been undertaken for diverse brain disorders.

## Highlights

• NIR light can penetrate the head and reach the brain.
• NIR is absorbed by cytochrome c oxidase in mitochondria.
• Heat-gated ion channels also play a role.
• Increased blood flow, energy, neuroprotection, less inflammation, brain repair
• Can treat traumatic (stroke, TBI), neurodegenerative and psychiatric diseases

## Graphical abstract

**Figure:**
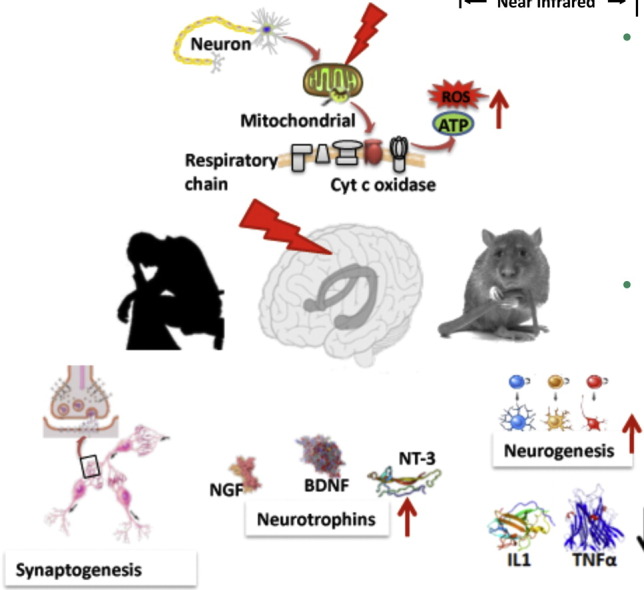

## 1 Introduction

Photobiomodulation (PBM) as it is known today (the beneficial health benefits of light therapy had been known for some time before), was accidently discovered in 1967, when Endre Mester from Hungary attempted to repeat an experiment recently published by McGuff in Boston, USA [1]. McGuff had used a beam from the recently discovered ruby laser [2], to destroy a cancerous tumor that had been experimentally implanted into a laboratory rat. However (unbeknownst to Mester) the ruby laser that had been built for him, was only a tiny fraction of the power of the laser that had previously been used by McGuff. However, instead of curing the experimental tumors with his low-powered laser, Mester succeeded in stimulating hair regrowth and wound healing in the rats, in the sites where the tumors had been implanted [3], [4]. This discovery led to a series of papers describing what Mester called “laser biostimulation”, and soon became known as “low level laser therapy” (LLLT) [5], [6], [7].

LLLT was initially primarily studied for stimulation of wound healing, and reduction of pain and inflammation in various orthopedic conditions such as tendonitis, neck pain, and carpal tunnel syndrome [8]. The advent of light emitting diodes (LED) led to LLLT being renamed as “low level light therapy”, as it became more accepted that the use of coherent lasers was not absolutely necessary, and a second renaming occurred recently [9] when the term PBM was adopted due to uncertainties in the exact meaning of “low level”.

## 2 Mechanisms of action of photobiomodulation

### 2.1 Mitochondria and cytochrome c oxidase

The most well studied mechanism of action of PBM centers around cytochrome c oxidase (CCO), which is unit four of the mitochondrial respiratory chain, responsible for the final reduction of oxygen to water using the electrons generated from glucose metabolism [10]. The theory is that CCO enzyme activity may be inhibited by nitric oxide (NO) (especially in hypoxic or damaged cells). This inhibitory NO can be dissociated by photons of light that are absorbed by CCO (which contains two heme and two copper centers with different absorption spectra) [11]. These absorption peaks are mainly in the red (600–700 nm) and near-infrared (760–940 nm) spectral regions. When NO is dissociated, the mitochondrial membrane potential is increased, more oxygen is consumed, more glucose is metabolized and more ATP is produced by the mitochondria.

### 2.2 Reactive oxygen species, nitric oxide, blood flow

It has been shown that there is a brief increase in reactive oxygen species (ROS) produced in the mitochondria when they absorb the photons delivered during PBM. The idea is that this burst of ROS may trigger some mitochondrial signaling pathways leading to cytoprotective, anti-oxidant and anti-apoptotic effects in the cells [12]. The NO that is released by photodissociation acts as a vasodilator as well as a dilator of lymphatic flow. Moreover NO is also a potent signaling molecule and can activate a number of beneficial cellular pathways [13]. Fig. 2 illustrates these mechanisms.

### 2.3 Light sensitive ion channels and calcium

It is quite clear that there must be some other type of photoacceptor, in addition to CCO, as is clearly demonstrated by the fact that wavelengths substantially longer than the red/NIR wavelengths discussed above, can also produce beneficial effects is some biological scenarios. Wavelengths such as 980 nm [14], [15], 1064 nm laser [16], and 1072 nm LED [17], and even broad band IR light [18] have all been reported to carry out PBM type effects. Although the photoacceptor for these wavelengths has by no means been conclusively identified, the leading hypothesis is that it is primarily water (perhaps nanostructured water) located in heat or light sensitive ion channels. Clear changes in intracellular calcium can be observed, that could be explained by light-mediated opening of calcium ion channels, such as members of the transient receptor potential (TRP) super-family [19]. TRP describes a large family of ion channels typified by TRPV1, recently identified as the biological receptor for capsaicin (the active ingredient in hot chili peppers) [20]. The biological roles of TRP channels are multifarious, but many TRP channels are involved in heat sensing and thermoregulation [21].

### 2.4 Signaling mediators and activation of transcription factors

Most authors suggest that the beneficial effects of tPBM on the brain can be explained by increases in cerebral blood flow, greater oxygen availability and oxygen consumption, improved ATP production and mitochondrial activity [22], [23], [24]. However there are many reports that a brief exposure to light (especially in the case of experimental animals that have suffered some kind of acute injury or traumatic insult) can have effects lasting days, weeks or even months [25]. This long-lasting effect of light can only be explained by activation of signaling pathways and transcription factors that cause changes in protein expression that last for some considerable time. The effects of PBM on stimulating mitochondrial activity and blood flow is of itself, unlikely to explain long-lasting effects. A recent review listed no less than fourteen different transcription factors and signaling mediators, that have been reported to be activated after light exposure [10].

**Fig. 1:**
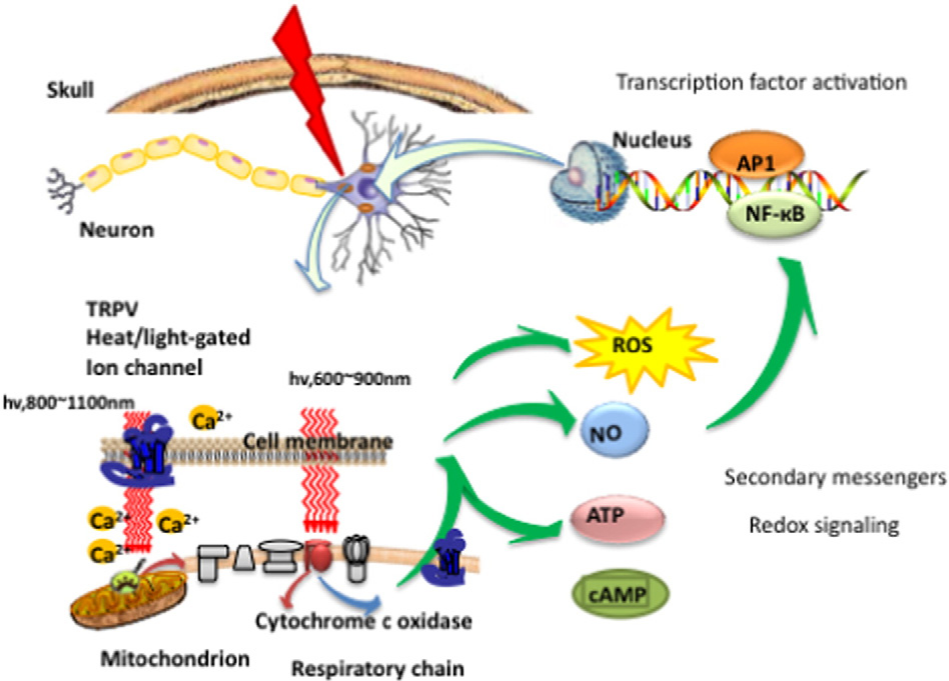
Molecular and intracellular mechanisms of transcranial low level laser (light) or photobiomodulation. AP1, activator protein 1; ATP, adenosine triphosphate; Ca^2 +,^ calcium ions; cAMP, cyclic adenosine monophosphate; NF-kB, nuclear factor kappa B; NO, nitric oxide; ROS, reactive oxygen species; TRPV, transient receptor potential vanilloid.

Fig. 1 illustrates two of the most important molecular photoreceptors or chromophores (cytochrome c oxidase and heat-gated ion channels) inside neuronal cells that absorb photons that penetrate into the brain. The signaling pathways and activation of transcription factors lead to the eventual effects of PBM in the brain.

**Fig. 2:**
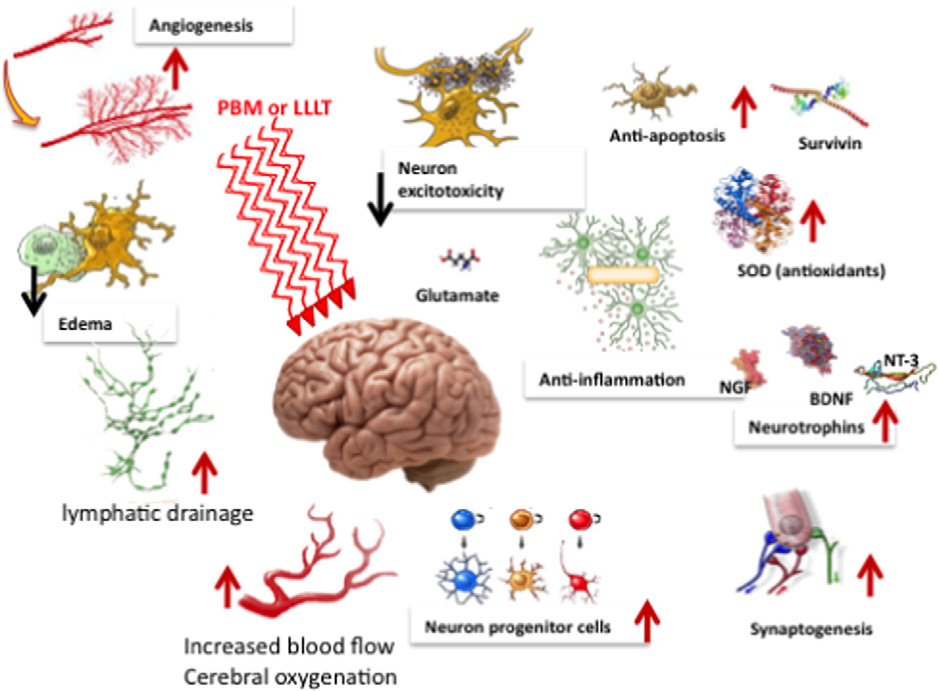
Tissue specific processes that occur after PBM and benefit a range of brain disorders. BDNF, brain-derived neurotrophic factor; LLLT, low level light therapy; NGF, nerve growth factor; NT-3, neurotrophin 3; PBM, photobiomodulation; SOD, superoxide dismutase.

Fig. 2 illustrates some more tissue specific mechanisms that lead on from the initial photon absorption effects explained in Fig. 1. A wide variety of processes can occur that can benefit a correspondingly wide range of brain disorders. These processes can be divided into short-term stimulation (ATP, blood flow, lymphatic flow, cerebral oxygenation, less edema). Another group of processes center around neuroprotection (upregulation of anti-apoptotic proteins, less excitotoxity, more antioxidants, less inflammation). Finally a group of processes that can be grouped under “help the brain to repair itself” (neurotrophins, neurogenesis and synaptogenesis).

### 2.5 Biphasic dose response and effect of coherence

The biphasic dose response (otherwise known as hormesis, and reviewed extensively by Calabrese et al. [26]) is a fundamental biological law describing how different biological systems can be activated or stimulated by low doses of any physical insult or chemical substance, no matter how toxic or damaging this insult may be in large doses. The most well studied example of hormesis is that of ionizing radiation, where protective mechanisms are induced by very low exposures, that can not only protect against subsequent large doses of ionizing radiation, but can even have beneficial effects against diseases such as cancer using whole body irradiation [27].

There are many reports of PBM following a biphasic dose response (sometimes called obeying the Arndt-Schulz curve [28], [29]. A low dose of light is beneficial, but raising the dose produces progressively less benefit until eventually a damaging effect can be produced at very high light [30]. It is often said in this context that “more does not mean more”.

Another question that arises in the field of PBM is whether the coherent monochromatic lasers that were used in the original discovery of the effect, and whose use continued for many years, are superior to the rather recent introduction of LEDs, that are non-coherent and have a wider band-spread (generally 30 nm full-width half-maximum). Although there are one or two authors who continue to believe that coherent lasers are superior [31], most commentators feel that other parameters such as wavelength, power density, energy density and total energy are the most important determinants of efficacy [8].

## 3 Tissue optics, direct versus systemic effects, light sources

### 3.1 Light penetration into the brain

Due to the growing interest in PBM of the brain, several tissue optics laboratories have investigated the penetration of light of different wavelengths through the scalp and the skull, and to what depths into the brain this light can penetrate. This is an intriguing question to consider, because at present it is unclear exactly what threshold of power density in mW/cm2 is required in the b5rain to have a biological effect. There clearly must be a minimum value below which the light can be delivered for an infinite time without doing anything, but whether this is in the region of μW/cm^2^ or mW/cm^2^ is unknown at present.

Functional near-infrared spectroscopy (fNIRS) using 700–900 nm light has been established as a brain imaging technique that can be compared to functional magnetic resonance imaging (fMRI) [32]. Haeussinger et al. estimated that the mean penetration depth (5% remaining intensity) of NIR light through the scalp and skull was 23:6 + 0:7 mm [33]. Other studies have found comparable results with variations depending on the precise location on the head and wavelength [34], [35].

Jagdeo et al. [36] used human cadaver heads (skull with intact soft tissue) to measure penetration of 830 nm light, and found that penetration depended on the anatomical region of the skull (0.9% at the temporal region, 2.1% at the frontal region, and 11.7% at the occipital region). Red light (633 nm) hardly penetrated at all. Tedord et al. [37] also used human cadaver heads to compare penetration of 660 nm, 808 nm, and 940 nm light. They found that 808 nm light was best and could reach a depth in the brain of 40–50 mm. Lapchak et al. compared the transmission of 810 nm light through the skulls of four different species, and found mouse transmitted 40%, while for rat it was 21%, rabbit it was 11.3 and for human skulls it was only 4.2% [38]. Pitzschke and colleagues compared penetration of 670 nm and 810 nm light into the brain when delivered by a transcranial or a transphenoidal approach, and found that the best combination was 810 nm delivered transphenoidally [39]. In a subsequent study these authors compared the effects of storage and processing (frozen or formalin-fixed) on the tissue optical properties of rabbit heads [40]. Yaroslavsky et al. examined light penetration of different wavelengths through different parts of the brain tissue (white brain matter, gray brain matter, cerebellum, and brainstem tissues, pons, thalamus). Best penetration was found with wavelengths between 1000 and 1100 nm [41].

Henderson and Morries found that between 0.45% and 2.90% of 810 nm or 980 nm light penetrated through 3 cm of scalp, skull and brain tissue in ex vivo lamb heads [42].

### 3.2 Systemic effects

It is in fact very likely that the beneficial effects of PBM on the brain cannot be entirely explained by penetration of photons through the scalp and skull into the brain itself. There have been some studies that have explicitly addressed this exact issue. In a study of PBM for Parkinson's disease in a mouse model [43]. Mitrofanis and colleagues compared delivering light to the mouse head, and also covered up the head with aluminum foil so that they delivered light to the remainder of the mouse body. They found that there was a highly beneficial effect on neurocognitive behavior with irradiation to the head, but nevertheless there was also a statistically significant (although less pronounced benefit, referred to by these authors as an ‘abscopal effect”) when the head was shielded from light [44]. Moreover Oron and co-workers [45] have shown that delivering NIR light to the mouse tibia (using either surface illumination or a fiber optic) resulted in improvement in a transgenic mouse model of Alzheimer's disease (AD). Light was delivered weekly for 2 months, starting at 4 months of age (progressive stage of AD). They showed improved cognitive capacity and spatial learning, as compared to sham-treated AD mice. They proposed that the mechanism of this effect was to stimulate c-kit-positive mesenchymal stem cells (MSCs) in autologous bone marrow (BM) to enhance the capacity of MSCs to infiltrate the brain, and clear β-amyloid plaques [46]. It should be noted that the calvarial bone marrow of the skull contains substantial numbers of stem cells [47].

### 3.3 Laser acupuncture

Laser acupuncture is often used as an alternative or as an addition to traditional Chinese acupuncture using needles [48]. Many of the applications of laser acupuncture have been for conditions that affect the brain [49] such as Alzheimer's disease [50] and autism [51] that have all been investigated in animal models. Moreover laser acupuncture has been tested clinically [52].

### 3.4 Light sources

A wide array of different light sources (lasers and LEDs) have been employed for tPBM. One of the most controversial questions which remains to be conclusively settled, is whether a coherent monochromatic laser is superior to non-coherent LEDs typically having a 30 nm band-pass (full width half maximum). Although wavelengths in the NIR region (800–1100 nm) have been the most often used, red wavelengths have sometimes been used either alone, or in combination with NIR. Power levels have also varied markedly from Class IV lasers with total power outputs in the region of 10 W [53], to lasers with more modest power levels (circa 1 W). LEDs can also have widely varying total power levels depending on the size of the array and the number and power of the individual diodes. Power densities can also vary quite substantially from the Photothera laser [54] and other class IV lasers , which required active cooling (~ 700 mW/cm^2^) to LEDs in the region of 10–30 mW/cm^2^.

### 3.5 Usefulness of animal models when testing tPBM for brain disorders

One question that is always asked in biomedical research, is how closely do the laboratory models of disease (which are usually mice or rats) mimic the human disease for which new treatments are being sought? This is no less critical a question when the areas being studied include brain disorders and neurology. There now exist a plethora of transgenic mouse models of neurological disease [55], [56]. However in the present case, where the proposed treatment is almost completely free of any safety concerns, or any reported adverse side effects, it can be validly questioned as to why the use of laboratory animal models should be encouraged. Animal models undoubtedly have disadvantages such as failure to replicate all the biological pathways found in human disease, difficulty in accurately measuring varied forms of cognitive performance, small size of mice and rats compared to humans, short lifespan affecting the development of age related diseases, and lack of lifestyle factors that adversely affect human diseases. Nevertheless, small animal models are less expensive, and require much less time and effort to obtain results than human clinical trials, so it is likely they will continue to be used to test tPBM for the foreseeable future.

## 4 PBM for stroke

### 4.1 Animal models

Perhaps the most well-investigated application of PBM to the brain, lies in its possible use as a treatment for acute stroke [57]. Animal models such as rats and rabbits, were first used as laboratory models, and these animals had experimental strokes induced by a variety of methods and were then treated with light (usually 810 nm laser) within 24 h of stroke onset [58]. In these studies intervention by tLLLT within 24 h had meaningful beneficial effects. For the rat models, stroke was induced by middle cerebral artery occlusion (MCAO) via an insertion of a filament into the carotid artery or via craniotomy [59], [60]. Stroke induction in the “rabbit small clot embolic model” (RSCEM) was by injection of a preparation of small blood clots (made from blood taken from a second donor rabbit) into a catheter placed in the right internal carotid artery [61]. These studies and the treatments and results are listed in Table 1.

CW, continuous wave; LLLT, low level light therapy; MCAO, middle cerebral artery occlusion; NOS, nitric oxide synthase; RSCEM, rabbit small clot embolic model; TGFβ1, transforming growth factor β1.

### 4.2 Clinical trials for acute stroke

Treatment of acute stroke was addressed in a series of three clinical trials called “Neurothera Effectiveness and Safety Trials” (NEST-1 [65], NEST-2 [66], and NEST-3 [67]) using an 810 nm laser applied to the shaved head within 24 h of patients suffering an ischemic stroke. The first study, NEST-1, enrolled 120 patients between the ages of 40 to 85 years of age with a diagnosis of ischemic stroke involving a neurological deficit that could be measured. The purpose of this first clinical trial was to demonstrate the safety and effectiveness of laser therapy for stroke within 24 h [65]. tPBM significantly improved outcome in human stroke patients, when applied at ~ 18 h post-stroke, over the entire surface of the head (20 points in the 10/20 EEG system) regardless of stroke [65]. Only one laser treatment was administered, and 5 days later, there was significantly greater improvement in the Real- but not in the Sham-treated group (*p* < 0.05, NIH Stroke Severity Scale). This significantly greater improvement was still present at 90 days post-stroke, where 70% of the patients treated with Real-LLLT had a successful outcome, while only 51% of Sham-controls did. The second clinical trial, NEST-2, enrolled 660 patients, aged 40 to 90, who were randomly assigned to one of two groups (331 to LLLT, 327 to sham) [68]. Beneficial results (*p* < 0.04) were found for the moderate and moderate-severe (but not for the severe) stroke patients, who received the Real laser protocol [68]. These results suggested that the overall severity of the individual stroke should be taken into consideration in future studies, and very severe patients are unlikely to recover with any kind of treatment. The last clinical trial, NEST-3, was planned for 1000 patients enrolled. Patients in this study were not to receive tissue plasminogen activator, but the study was prematurely terminated by the DSMB for futility (an expected lack of statistical significance) [67]. NEST-1 was considered successful, even though as a phase 1 trial, it was not designed to show efficacy. NEST-2 was partially successful when the patients were stratified, to exclude very severe strokes or strokes deep within the brain [66]. There has been considerable discussion in the scientific literature on precisely why the NEST-3 trial failed [69]. Many commentators have wondered how could tPBM work so well in the first trial, in a sub-group in the second trial, and fail in the third trial. Lapchak's opinion is that the much thicker skull of humans compared to that of the other animals discussed above (mouse, rat and rabbit), meant that therapeutically effective amounts of light were unlikely to reach the brain [69]. Moreover the time between the occurrence of a stroke and initiation of the PBMT may be an important factor. There are reports in the literature that neuroprotection must be administered as soon as possible after a stroke [70], [71]. Furthermore, stroke trials in particular should adhere to the RIGOR (rigorous research) guidelines and STAIR (stroke therapy academic industry roundtable) criteria [72]. Other contributory causes to the failure of NEST-3 may have been included the decision to use only one single tPBM treatment, instead of a series of treatments. Moreover, the optimum brain areas to be treated in acute stroke remain to be determined. It is possible that certain areas of the brain that have sustained ischemic damage should be preferentially illuminated and not others.

### 4.3 Chronic stroke

Somewhat surprisingly, there have not as yet been many trials of PBM for rehabilitation of stroke patients with only the occasional report to date. Naeser reported in an abstract the use of tPBM to treat chronic aphasia in post-stroke patients [73]. Boonswang et al. [74] reported a single patient case in which PBM was used in conjunction with physical therapy to rehabilitate chronic stroke damage. However the findings that PBM can stimulate synaptogenesis in mice with TBI, does suggest that tPBM may have particular benefits in rehabilitation of stroke patients. Norman Doidge, in Toronto, Canada has described the use of PBM as a component of a neuroplasticity approach to rehabilitate chronic stroke patients [75].

## 5 PBM for traumatic brain injury (TBI)

### 5.1 Mouse and rat models

There have been a number of studies looking at the effects of PBM in animal models of TBI. Oron's group was the first [76] to demonstrate that a single exposure of the mouse head to a NIR laser (808 nm) a few hours after creation of a TBI lesion could improve neurological performance and reduce the size of the brain lesion. A weight-drop device was used to induce a closed-head injury in the mice. An 808 nm diode laser with two energy densities (1.2–2.4 J/cm^2^ over 2 min of irradiation with 10 and 20 mW/cm^2^) was delivered to the head 4 h after TBI was induced. Neurobehavioral function was assessed by the neurological severity score (NSS). There were no significant difference in NSS between the power densities (10 vs 20 mW/cm^2^) or significant differentiation between the control and laser treated group at early time points (24 and 48 h) post TBI. However, there was a significant improvement (27% lower NSS score) in the PBM group at times of 5 days to 4 weeks. The laser treated group also showed a smaller loss of cortical tissue than the sham group [76].

**Fig. 3:**
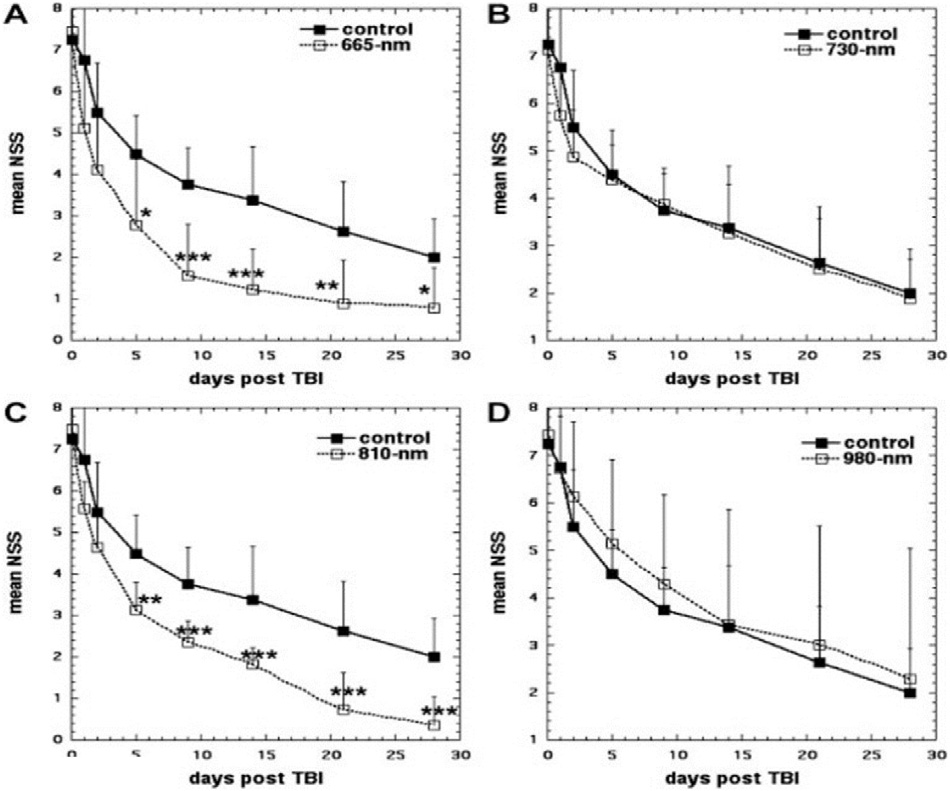
tPBM for TBI in a mouse model. Mice received a closed head injury and 4 hours later a single exposure of the head to one of four different lasers (36 J/cm^2^ delivered at 150 mW/cm^2^ over 4 min with spot size 1-cm diameter) [77]. A, 665 nm; B, 730 nm; C, 810 nm; D, 980 nm.

Hamblin's laboratory then went on (in a series of papers [76]) to show that 810 nm laser (and 660 nm laser) could benefit experimental TBI both in a closed head weight drop model [77], and also in controlled cortical impact model in mice [25]. Wu et al. [77] explored the effect that varying the laser wavelengths of LLLT had on closed-head TBI in mice. Mice were randomly assigned to LLLT treated group or to sham group as a control. Closed-head injury (CHI) was induced via a weight drop apparatus. To analyze the severity of the TBI, the neurological severity score (NSS) was measured and recorded. The injured mice were then treated with varying wavelengths of laser (665, 730, 810 or 980 nm) at an energy level of 36 J/cm^2^ at 4 h directed onto the scalp. The 665 nm and 810 nm groups showed significant improvement in NSS when compared to the control group at day 5 to day 28. Results are shown in Fig. 3. Conversely, the 730 and 980 nm groups did not show a significant improvement in NSS and these wavelengths did not produce similar beneficial effects as in the 665 nm and 810 nm LLLT groups [77]. The tissue chromophore cytochrome c oxidase (CCO) is proposed to be responsible for the underlying mechanism that produces the many PBM effects that are the byproduct of LLLT. COO has absorption bands around 665 nm and 810 nm while it has low absorption bands at the wavelength of 730 nm [78]. It should be noted that this particular study found that the 980 nm did not produce the same positive effects as the 665 nm and 810 nm wavelengths did; nevertheless previous studies did find that the 980 nm wavelength was an active one for LLLT. Wu et al. proposed that these dissimilar results may be due to the variance in the energy level, irradiance, etc. between the other studies and this particular study [77].

**Fig. 4:**
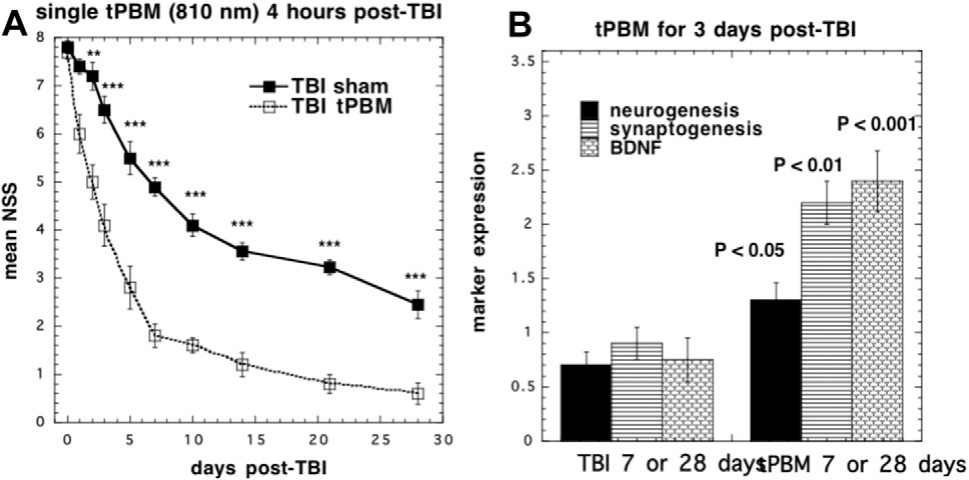
tPBM for controlled cortical impact TBI in a mouse model. (A) Mice received a single exposure (810 nm laser, 36 J/cm^2^ delivered at 50 mW/cm^2^ over 12 min) [121]. (B) Mice received 3 daily exposures starting 4 h post-TBI and were sacrificed after 7 or 28 days. BDNF and neurogenesis (BrdU) were increased at 7 days [81], while synaptogenesis was increased at 28 days [80].

Ando et al. [25] used the 810 nm wavelength laser parameters from the previous study and varied the pulse modes of the laser in a mouse model of TBI. These modes consisted of either pulsed wave at 10 Hz or at 100 Hz (50% duty cycle) or continuous wave laser. For the mice, TBI was induced with a controlled cortical impact device via open craniotomy. A single treatment with an 810 nm Ga-Al-As diode laser with a power density of 50 mW/m^2^ and an energy density of 36 J/cm^2^ was given via tLLLT to the closed head in mice for a duration of 12 min at 4 h post CCI. At 48 h to 28 days post TBI, all laser treated groups had significant decreases in the measured neurological severity score (NSS) when compared to the control (Fig. 4A). Although all laser treated groups had similar NSS improvement rates up to day 7, the PW 10 Hz group began to show greater improvement beyond this point as seen in Fig. 4. At day 28, the forced swim test for depression and anxiety was used and showed a significant decrease in the immobility time for the PW 10 Hz group. In the tail suspension test which measures depression and anxiety, there was also a significant decrease in the immobility time at day 28, and this time also at day 1, in the PW 10 Hz group.

Studies using immunofluorescence of mouse brains showed that tPBM increased neuroprogenitor cells in the dentate gyrus (DG) and subventricular zone at 7 days after the treatment [79]. The neurotrophin called brain derived neurotrophic factor (BDNF) was also increased in the DG and SVZ at 7 days , while the marker (synapsin-1) for synaptogenesis and neuroplasticity was increased in the cortex at 28 days but not in the DG, SVZ or at 7 days [80] (Fig. 4B). Learning and memory as measured by the Morris water maze was also improved by tPBM [81]. Whalen's laboratory [82] and Whelan's laboratory [83] also successfully demonstrated therapeutic benefits of tPBM for TBI in mice and rats respectively.

Zhang et al. [84] showed that secondary brain injury occurred to a worse degree in mice that had been genetically engineered to lack “Immediate Early Response” gene X-1 (IEX-1) when exposed to a gentle head impact (this injury is thought to closely resemble mild TBI in humans). Exposing IEX-1 knockout mice to LLLT 4 h post injury, suppressed proinflammatory cytokine expression of interleukin (IL)-Iβ and IL-6, but upregulated TNF-α. The lack of IEX-1 decreased ATP production, but exposing the injured brain to LLLT elevated ATP production back to near normal levels.

Dong et al. [85] even further improved the beneficial effects of PBM on TBI in mice, by combining the treatment with metabolic substrates such as pyruvate and/or lactate. The goal was to even further improve mitochondrial function. This combinatorial treatment was able to reverse memory and learning deficits in TBI mice back to normal levels, as well as leaving the hippocampal region completely protected from tissue loss; a stark contrast to that found in control TBI mice that exhibited severe tissue loss from secondary brain injury.

### 5.2 TBI in humans

Margaret Naeser and collaborators have tested PBM in human subjects who had suffered TBI in the past [86]. Many sufferers from severe or even moderate TBI, have very long lasting and even life-changing sequelae (headaches, cognitive impairment, and difficulty sleeping) that prevent them working or living any kind or normal life. These individuals may have been high achievers before the accident that caused damage to their brain [87]. Initially Naeser published a report [88] describing two cases she treated with PBM applied to the forehead twice a week. A 500 mW continuous wave LED source (mixture of 660 nm red and 830 nm NIR LEDs) with a power density of 22.2 mW/cm^2^ (area of 22.48 cm^2^), was applied to the forehead for a typical duration of 10 min (13.3 J/cm^2^). In the first case study the patient reported that she could concentrate on tasks for a longer period of time (the time able to work at a computer increased from 30 min to 3 h). She had a better ability to remember what she read, decreased sensitivity when receiving haircuts in the spots where LLLT was applied, and improved mathematical skills after undergoing LLLT. The second patient had statistically significant improvements compared to prior neuropsychological tests after 9 months of treatment. The patient had a 2 standard deviation (SD) increase on tests of inhibition and inhibition accuracy (9th percentile to 63rd percentile on the Stroop test for executive function and a 1 SD increase on the Wechsler Memory scale test for the logical memory test (83rd percentile to 99th percentile) [89].

Naeser et al. then went on to report a case series of a further eleven patients [90]. This was an open protocol study that examined whether scalp application of red and near infrared (NIR) light could improve cognition in patients with chronic, mild traumatic brain injury (mTBI). This study had 11 participants ranging in age from 26 to 62 (6 males, 5 females) who suffered from persistent cognitive dysfunction after mTBI. The participants' injuries were caused by motor vehicle accidents, sports related events and for one participant, an improvised explosive device (IED) blast. tLLLT consisted of 18 sessions (Monday, Wednesday, and Friday for 6 weeks) and commenced anywhere from 10 months to 8 years post-TBI. A total of 11 LED clusters (5.25 cm in diameter, 500 mW, 22.2 mW/cm^2^, 13 J/cm^2^) were applied for about 10 min per session (5 or 6 LED placements per set, Set A and then Set B, in each session). Neuropsychological testing was performed pre-LED application and 1 week, 1 month and 2 months after the final treatment. Naeser and colleagues found that there was a significant positive linear trend observed for the Stroop Test for executive function, in trial 2 inhibition (*p* = 0.004); Stroop, trial 4 inhibition switching (*p* = 0.003); California Verbal Learning Test (CVLT)-II, total trials 1–5 (p = 0.003); CVLT-II, long delay free recall (*p* = 0.006). Improved sleep and fewer post-traumatic stress disorder (PTSD) symptoms, if present beforehand, were observed after treatment. Participants and family members also reported better social function and a better ability to perform interpersonal and occupational activities. Although these results were significant, further placebo-controlled studies will be needed to ensure the reliability of this these data [90].

Henderson and Morries [91] used a high-power NIR laser (10–15 W at 810 and 980 nm) applied to the head to treat a patient with moderate TBI. The patient received 20 NIR applications over a 2-month period. They carried out anatomical magnetic resonance imaging (MRI) and perfusion single-photon emission computed tomography (SPECT). The patient showed decreased depression, anxiety, headache, and insomnia, whereas cognition and quality of life improved, accompanied by changes in the SPECT imaging.

## 6 PBM for Alzheimer's disease (AD)

### 6.1 Animal models

There was a convincing study [92] carried out in an AβPP transgenic mouse of AD. tPBM (810 nm laser) was administered at different doses 3 times/week for 6 months starting at 3 months of age. The numbers of Aβ plaques were significantly reduced in the brain with administration of tPBM in a dose-dependent fashion. tPBM mitigated the behavioral effects seen with advanced amyloid deposition and reduced the expression of inflammatory markers in the transgenic mice. In addition, TLT showed an increase in ATP levels, mitochondrial function, and c-fos expression suggesting that there was an overall improvement in neurological function.

### 6.2 Humans

**Fig. 5:**
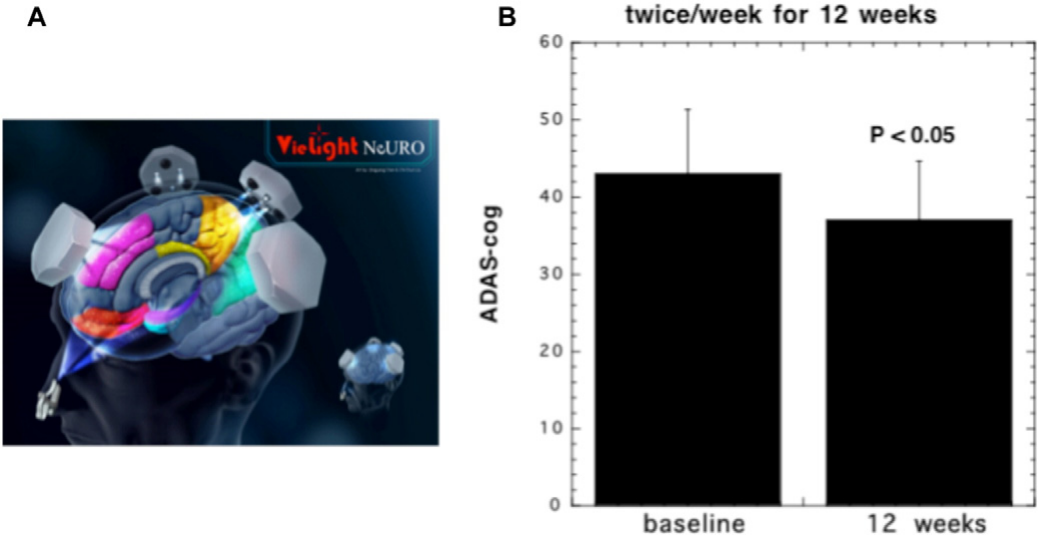
tPBM for Alzheimer's disease. (A) Nineteen patients were randomized to receive real or sham tPBM (810 nm LED, 24.6 J/cm^2^ at 41 mW/cm^2^). (B) Significant decline in ADAS-cog (improved cognitive performance) in real but not sham (unpublished data).

There has been a group of investigators in Northern England who have used a helmet built with 1072 nm LEDs to treat AD, but somewhat surprisingly no peer-reviewed publications have described this approach [93]. However a small pilot study (19 patients) that took the form of a randomized placebo-controlled trial investigated the effect of the Vielight Neuro system (see Fig. 5A) (a combination of tPBM and intranasal PBM) on patients with dementia and mild cognitive impairment [94]. This was a controlled single blind pilot study in humans to investigate the effects of PBM on memory and cognition. 19 participants with impaired memory/cognition were randomized into active and sham treatments over 12 weeks with a 4-week no-treatment follow-up period. They were assessed with MMSE and ADAS-cog scales. The protocol involved in-clinic use of a combined transcranial-intranasal PBM device; and at-home use of an intranasal-only PBM device and participants/ caregivers noted daily experiences in a journal. Active participants with moderate to severe impairment (MMSE scores 5–24) showed significant improvements (5-points MMSE score) after 12 weeks. There was also a significant improvement in ADAS-cog scores (see Fig. 5B). They also reported better sleep, fewer angry outbursts and decreased anxiety and wandering. Declines were noted during the 4-week no-treatment follow-up period. Participants with mild impairment to normal (MMSE scores of 25 to 30) in both the active and sham sub-groups showed improvements. No related adverse events were reported.

An interesting paper from Russia [95] described the use of intravascular PBM to treat 89 patients with AD who received PBM (46 patients) or standard treatment with memantine and rivastigmine (43 patients). The PBM consisted of threading a fiber-optic through a cathéter in the fémoral artery and advancing it to the distal site of the anterior and middle cerebral arteries and delivering 20 mW of red laser for 20–40 min. The PBM group had improvement in cerebral microcirculation leading to permanent (from 1 to 7 years) reduction in dementia and cognitive recovery.

## 7 Parkinson's disease

The majority of studies on PBM for Parkinson's disease have been in animal models and have come from the laboratory of John Mitrofanis in Australia [96]. Two basic models of Parkinson's disease were used. The first employed administration of the small molecule (MPTP or 1-methyl-4-phenyl-1,2,3,6-tetrahydropyridine) to mice [97]. MPTP was discovered as an impurity in an illegal recreational drug to cause Parkinson's like symptoms (loss of substantia nigra cells) in young people who had taken this drug [98]. Mice were treated with tPBM (670-nm LED, 40 mW/cm^2^, 3.6 J/cm^2^) 15 min after each MPTP injection repeated 4 times over 30 h. There were significantly more (35%–45%) dopaminergic cells in the brains of the tPBM treated mice [97]. A subsequent study showed similar results in a chronic mouse model of MPTP-induced Parkinson's disease [99]. They repeated their studies in another mouse model of Parkinson's disease, the tau transgenic mouse strain (K3) that has a progressive degeneration of dopaminergic cells in the substantia nigra pars compacta (SNc) [100]. They went on to test a surgically implanted intracranial fiber designed to deliver either 670 nm LED (0.16 mW) or 670 nm laser (67 mW) into the lateral ventricle of the brain in MPTP-treated mice [101]. Both low power LED and high power laser were effective in preserving SNc cells, but the laser was considered to be unsuitable for long-term use (6 days) due to excessive heat production. As mentioned above, these authors also reported a protective effect of abscopal light exposure (head shielded) in this mouse model [43]. Recently this group has tested their implanted fiber approach in a model of Parkinson's disease in adult Macaque monkeys treated with MPTP [102]. Clinical evaluation of Parkinson's symptoms (posture, general activity, bradykinesia, and facial expression) in the monkeys were improved at low doses of light (24 J or 35 J) compared to high doses (125 J) [103].

The only clinical report of PBM for Parkinson's disease in humans was an abstract presented in 2010 [104]. Eight patients between 18 and 80 years with late stage PD participated in a non-controlled, non-randomized study. Participants received tPBM treatments of the head designed to deliver light to the brain stem, bilateral occipital, parietal, temporal and frontal lobes, and treatment along the sagittal suture. A Visual Analog Scale (VAS), was used to record the severity of their symptoms of balance, gait, freezing, cognitive function, rolling in bed, and difficulties with speech pre-procedure and at study endpoint with 10 being most severe and 0 as no symptom. Compared with baseline, all participants demonstrated a numerical improvement in the VAS from baseline to study endpoint. A statistically significant reduction in VAS rating for gait and cognitive function was observed with average mean change of —1.87 (*p* < 0.05) for gait and a mean reduction of —2.22 (*p* < 0.05) for cognitive function. Further, freezing and difficulty with speech ratings were significantly lower (mean reduction of 1.28 (*p* < 0.05) for freezing and 2.22 (*p* < 0.05) for difficulty with speech).

## 8 PBM for psychiatric disorders

### 8.1 Animal models

A common and well-accepted animal model of depression is called “chronic mild stress” [105]. After exposure to a series of chronic unpredictable mild stressors, animals develop symptoms seen in human depression, such as anhedonia (loss of the capacity to experience pleasure, a core symptom of major depressive disorder), weight loss or slower weight gain, decrease in locomotor activity, and sleep disorders [106]. Wu et al. used Wistar rats to show that after 5 weeks of chronic stress, application of tPBM 3 times a week for 3 weeks (810 nm laser, 100 Hz with 20% duty cycle, 120 J/cm^2^) gave significant improvement in the forced swimming test (FST) [107]. In a similar study Salehpour et al. [108] compared the effects of two different lasers (630 m nm at 89 mW/cm^2^, and 810 nm at 562 mW/cm^2^, both pulsed at 10 Hz, 50% duty cycle). The 810 nm laser proved better than the 630 nm laser in the FST, in the elevated plus maze and also reduced blood cortisol levels.

### 8.2 Depression and anxiety

**Fig. 6:**
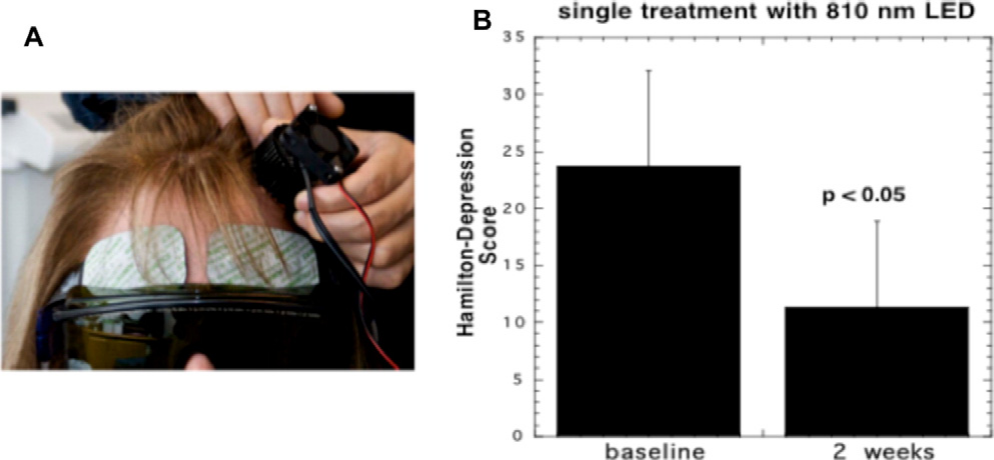
tPBM for major depression and anxiety in humans. (A) Ten patients received a single exposure to the forehead (810 LED, 60 J/cm^2^ delivered at 250 mW/cm^2^). (B) Mean Hamilton score for depression at baseline and at two weeks post-treatment [109].

The first clinical study in depression and anxiety was published by Schiffer et al. in 2009 [109]. They used a fairly small area 1 W 810 nm LED array (see Fig. 6A) applied to the forehead in patients with major depression and anxiety. They found improvements in the Hamilton depression rating scale (HAM-D) (see Fig. 6B), and the Hamilton anxiety rating scale (HAM-A), 2 weeks after a single treatment. They also found increases in frontal pole regional cerebral blood flow (rCBF) during the light delivery using a commercial NIR spectroscopy device. Cassano and co-workers [110] used tPBM with an 810 nm laser (700 mW/cm^2^ and a fluence of 84  J/cm^2^ delivered per session for 6 sessions in patients with major depression. Baseline mean HAM-D17 scores decreased from 19.8 ± 4.4 (SD) to 13 ± 5.35 (SD) after treatment (*p* = 0.004).

## 9 Cognitive enhancement

From what we have seen above, it need come as no surprise, to learn that there are several reports about cognitive enhancement in normal people or healthy animals using PBM. The first report was in middle aged (12 months) CD1 female mice [111]. Exposure of the mice to 1072 nm LED arrays led to improved performance in a 3D maze compared to sham treated age-matched controls. Francisco Gonzalez-Lima at the University of Texas Austin, has worked in this area for some time [112]. Working in rats they showed that transcranial PBM (9 mW/cm^2^ with 660 nm LED array) induced a dose-dependent increase in oxygen consumption of 5% after 1 J/cm^2^ and 16% after 5 J/cm^2^ [113]. They also found that tPBM reduced fear renewal and prevented the reemergence of extinguished conditioned fear responses [113]. In normal human volunteers they used transcranial PBM (1064 nm laser, 60 J/cm^2^ at 250 mW/cm^2^) delivered to the forehead in a placebo-controlled, randomized study, to influence cognitive tasks related to the prefrontal cortex, including a psychomotor vigilance task (PVT), a delayed match-to-sample (DMS) memory task, and the positive and negative affect schedule (PANAS-X) to show improved mood [16]. Subsequent studies in normal humans showed that tPBM with 1064 nm laser could improve performance in the Wisconsin Card Sorting Task (considered the gold standard test for executive function) [114]. They also showed that tPBM to the right forehead (but not the left forehead) had better effects on improving attention bias modification (ABM) in humans with depression [115].

A study by Salgado et al. used transcranial LED PBM on cerebral blood flow in healthy elderly women analyzed by transcranial Doppler ultrasound (TCD) of the right and left middle cerebral artery and basilar artery. Twenty-five non-institutionalized elderly women (mean age 72 years old), with cognitive status > 24, were assessed using TCD before and after transcranial LED therapy. tPBM (627 nm, 70 mW/cm^2^, 10 J/cm^2^) was performed at four points of the frontal and parietal region for 30 s each twice a week for 4 weeks. There was a significant increase in the systolic and diastolic velocity of the left middle cerebral artery (25 and 30%, respectively) and the basilar artery (up to 17 and 25%), as well as a decrease in the pulsatility index and resistance index values of the three cerebral arteries analyzed [116].

## 10 Conclusion

Many investigators believe that PBM for brain disorders will become one of the most important medical applications of light therapy in the coming years and decades. Despite the efforts of “Big Pharma”, prescription drugs for psychiatric disorders are not generally regarded very highly (either by the medical profession or by the public), and many of these drugs perform little better than placebos in different trials, and moreover can also have major side-effects [117]. Moreover it is well accepted that with the overall aging of the general population, together with ever lengthening life spans, that dementia, Alzheimer's, and Parkinson's diseases will become a global health problem [118], [119]. Even after many years of research, no drug has yet been developed to benefit these neurodegenerative disorders. A similar state of play exists with drugs for stroke (with the exception of clot-busting enzymes) and TBI. New indications for tPBM such as global ischemia (brain damage after a heart attack), post-operative cognitive dysfunction [120], and neurodevelopmental disorders such as autism spectrum disorder may well emerge. Table 2 shows the wide range of brain disorders and diseases that may eventually be treated by some kind of tPBM, whether that be an office/clinic based procedure or a home-use based device. If inexpensive LED helmets can be developed and successfully marketed as home use devices, then we are potentially in a position to benefit large numbers of patients (to say nothing of healthy individuals). Certainly the advent of the Internet has made it much easier for knowledge about this kind of home treatment to spread (almost by word of mouth so to speak).

## Conflict of interest statement

The author declares no conflict of interest.

## Transparency document

Transparency document. Image 1

## Figures and Tables

**Table 1:** Reports of transcranial LLLT used for stroke in animal models.

Subject	Stroke model	Parameters	Effect	References
Rat	MCAO	660 nm; 8.8 mW; 2.64 J/cm^2^; pulse frequency of 10 kHz; laser applied at cerebrum at 1, 5 and 10 min	Suppression of NOS activity and up regulation of TGF-β1	[59]
Rat	MCAO	808 nm; 7.5 mW/cm^2^; 0.9 J/cm^2^; 3.6 J/cm^2^ at cortical surface; CW and pulse wave at 70 Hz, 4 mm diameter	Administration of LLLT 24 h after stroke onset induced functional benefit and neurogenesis induction	[60]
Rabbit	RSCEM	808 nm ± 5 nm; 7.5 W/cm^2^, 2 min duration 3 h after stroke and 25 mW/cm^2^ 10 min duration 1 or 6 h after stroke	Improved behavioral performance and durable effect after LLLT within 6 h from stroke onset	[62]
Rat	MCAO	808 nm; 0.5 mW/cm^2^; 0.9 J/cm^2^ on brain 3 mm dorsal to the eye and 2 mm anterior to the ear	LLLT applied at different locations on the skull improved neurological function after acute stroke	[63]
Rabbit	RSCEM	808 nm; 7.5 mW/cm^2^; 0.9 J/cm^2^; 3.6 J/cm^2^ at cortical surface; CW; 300 min; pulse at 1 kHz, 2 min at 100 Hz	LLLT administered 6 h after embolic stroke resulted in clinical improvements in rabbits	[64]

**Table 2:** List of brain disorders that may in principle be treated by tPBM.

Type of brain disorder			
Traumatic	Neurodegenerative	Psychiatric	Neurodevelopmental
Acute stroke	Alzheimer's disease	Depression (major, bipolar, suicidal ideation)	Autism (autism spectrum disorder)
Chronic stroke	Parkinson's disease	Psychosis (schizophrenia)	Attention deficit hyperactivity disorder (ADHD)
Acute TBI	Other dementias (vascular, Lewy bodies, frontotemporal)	Post traumatic stress disorder (PTSD)	
Chronic TBI	Chronic traumatic encephalopathy	Addiction	
Global ischemia	Amyotrophic lateral sclerosis (Lou Gehrig's disease)	Insomnia	
Coma (vegetative state)	Primary progressive aphasia		
Birth trauma (neonatal stroke)	Prion diseases (Creutzfeldt-Jakob)		
“Chemo-brain”	Huntington's disease		
